# A Spanish Validation of the Canadian Adolescent Gambling Inventory (CAGI)

**DOI:** 10.3389/fpsyg.2017.00177

**Published:** 2017-02-07

**Authors:** Susana Jiménez-Murcia, Roser Granero, Randy Stinchfield, Joël Tremblay, Amparo del Pino-Gutiérrez, Laura Moragas, Lamprini G. Savvidou, Fernando Fernández-Aranda, Neus Aymamí, Mónica Gómez-Peña, Salomé Tárrega, Katarina Gunnard, Virginia Martín-Romera, Trevor Steward, Gemma Mestre-Bach, José M. Menchón

**Affiliations:** ^1^Pathological Gambling Unit, Department of Psychiatry, Bellvitge University Hospital – Institut d’Investigació Biomèdica de BellvitgeBarcelona Spain; ^2^Ciber Fisiopatología Obesidad y Nutrición, Instituto de Salud Carlos IIIMadrid, Spain; ^3^Department of Clinical Sciences, School of Medicine, University of BarcelonaBarcelona, Spain; ^4^Departament de Psicobiologia i Metodologia de les Ciències de la Salut, Universitat Autònoma de BarcelonaBarcelona, Spain; ^5^Department of Psychiatry, University of Minnesota Medical SchoolMinneapolis, MN, USA; ^6^Département de Psychoéducation, Université du Québec à Trois-RivièresTrois-Rivières, QC, Canada; ^7^Departament d’Infermeria de Salut Pública, Salut Mental i Maternoinfantil, Escola Universitària d’Infermeria, Universitat de BarcelonaBarcelona, Spain; ^8^Facultat de Psicologia, Universitat Autònoma de BarcelonaBarcelona, Spain; ^9^CIBER Salud Mental (CIBERSAM), Instituto de Salud Carlos IIIMadrid, Spain

**Keywords:** adolescence, CAGI, gambling disorder, youth, psychometric properties, validation

## Abstract

**Aims:** Large-scale epidemiological studies show a significant prevalence of gambling disorder (GD) during adolescence and emerging adulthood, and highlight the need to identify gambling-related behaviors at early ages. However, there are only a handful of screening instruments for this population and many studies measuring youth gambling problems use adult instruments that may not be developmentally appropriate. The aim of this study was to validate a Spanish version of the *Canadian Adolescent Gambling Inventory* (CAGI) among late adolescent and young adults and to explore its psychometric properties.

**Methods:** The sample (16–29 years old) included a clinical group (*n* = 55) with GD patients and a control group (*n* = 340).

**Results:** Exploratory factor analysis yielded one factor as the best model. This 24-item scale demonstrated satisfactory reliability (internal consistency, Cronbach’s alpha, α = 0.91), satisfactory convergent validity as measured by correlation with South Oaks Gambling Screen (*r* = 0.74), and excellent classification accuracy (AUC = 0.99; sensitivity = 0.98; and specificity = 0.99).

**Conclusion:** Our results provide empirical support for our validation of the Spanish version of the CAGI. We uphold that the Spanish CAGI can be used as a brief, reliable, and valid instrument to assess gambling problems in Spanish youth.

## Introduction

Gambling disorder (GD) is defined as persistent and recurrent problematic gambling behavior leading to clinically significant impairment or distress (American Psychiatric Association [APA], 2013). Age of onset varies widely between samples (Kessler et al., 2008), but a common trend toward gambling at younger ages has been identified in many countries (Volberg et al., 2010). It has been postulated that this may be a consequence of the expansion of gambling opportunities, the increased use of new technologies and the legalization of online gambling (Granero et al., 2014; Shin et al., 2014; Gainsbury et al., 2015). The onset of gambling behavior at an early age is an important risk factor for the rapid development of GD (Raylu and Oei, 2002; Jiménez-Murcia et al., 2009; Johansson et al., 2009; Lorains et al., 2011; Castrén et al., 2013) and is associated with other problematic behaviors, such as drug/alcohol abuse (Kessler et al., 2008) and eating disorders (Jiménez-Murcia et al., 2013; Von Ranson et al., 2013). Recent studies agree on the need for instruments to better assess the impact of exposure to gambling among young people (Balogh et al., 2013; Lee et al., 2014; Edgerton et al., 2015; Calado et al., 2016).

To date, it has been common practice to adapt instruments constructed for adult gambling assessment to measure gambling behavior and risks among adolescents (Dodig, 2013). This approach greatly reduces the reliability of research conducted on this population as items intended for adults fail to inquire about the effects of gambling on peer relationships in a developmentally appropriate manner (Edgren et al., 2016). Furthermore, the lack of empirical evidence regarding the validity of such adaptations, coupled with the fact that different studies use different adult instrument adaptations, makes it a challenge to reach meaningful conclusions regarding risk factors in this vulnerable population (Stinchfield, 2002; Derevensky et al., 2003). So far, no prevalence studies on underage gambling have been conducted in Spain, though other research suggests that a large number of adult Spanish gamblers began playing while they were minors (Jiménez-Murcia et al., 2014; Estevez et al., 2015).

To address these caveats, the Canadian Adolescent Gambling Inventory (CAGI) was specifically developed to measure gambling behavior among youths (Tremblay et al., 2010a). The content of the CAGI include: type of gambling activities, frequency of participation and time spent on each gambling activity, total money spent on gambling, high risk gambling behaviors and consequences of excessive gambling. The CAGI also provides a general measure of psychosocial consequences related to gambling. Despite its relatively rare use, the CAGI has been lauded for having a strong theoretical and methodological base, given that it provides a developmentally-adjusted evaluation of problem gambling behavior (e.g., socializing with groups that more intensely partake in games of chance) (Dodig, 2013). Thus far, two peer-reviewed studies have been published using this instrument, one in a community sample of Croatian adolescents (Ricijas et al., 2016) and another in a large sample of American college students (King et al., 2010). Therefore, the aim of the present study was to validate a Spanish version of the CAGI in a sample of young people and to assess its psychometric properties.

## Materials and Methods

### Participants

Two groups were recruited, a clinical and a control group. The clinical subsample included *n* = 55 young men (16–29 years old) who were diagnosed with GD and who were admitted to outpatient treatment at a specialized Gambling Disorder Unit at the Bellvitge University Hospital in Barcelona (Spain). These patients were assessed by experienced clinical psychologists and psychiatrists who made their diagnoses according to DSM-IV-TR criteria (American Psychiatric Association [APA], 2000) The control subsample included *n* = 340 participants (aged 17–29) recruited from the same university hospital setting. Unit staff confirmed that participants in the control group did not meet the DSM-IV-TR criteria for GD using the Stinchfield Diagnostic Questionnaire for Pathological Gambling (Stinchfield, 2003; Stinchfield et al., 2007; Jiménez-Murcia et al., 2009). The inclusion of the control group allowed us to test the accuracy-validity of the CAGI to identify the presence of GD [sensitivity, specificity, and other indexes based on receiver operating characteristics (ROC) methodology]. Recruitment of participants took place from March 2012 to September 2013.

### Instruments

#### Canadian Adolescent Gambling Inventory (CAGI; Tremblay et al., 2010a,b)

The CAGI is a self-report instrument that measures the adverse psychosocial consequences of gambling in adolescent populations. It is made up of two sections. The first section examines gambling participation in the past 3 months and includes 20 items to measure gambling frequency (using six-point response options) and the time spent gambling in a typical week, examining 19 different types of gambling activities. A final item in this section examines the amount of money and items of value lost because of gambling. The second section contains 24 items (using a four-point response option) that cover five domains: (a) gambling problem severity (nine items); (b) psychological consequences (six items); (c) social consequences (five items); (d) financial consequences (six items); and (e) loss of control (four items). The CAGI also includes a general problem severity subscale (GPSS), which consists of nine items distributed through the four CAGI subscales. The GPSS was developed as a gambling problem severity classification tool and yields a score ranging from 0 to 27. This final score gives a degree of global gambling severity and classifies the scores into three categories: (1) 0–1 no problem gambling (“green light”), (2) 2–5 low to moderate severity (“yellow light”), and (3) 6+ high severity (“red light”). The GPSS provided good classification accuracy for a cut-off point of 6 with sensitivity *Sensitivity* = 0.97 and *Specificity* = 0.93 (Tremblay et al., 2010a). Respondents in surveys utilizing CAGI provide replies on a four-element scale, with the format of the offered answer depending on the content of the question (never – sometimes – most of the time – almost always, or never – one to three times – four to six times – seven or more times).

#### South Oaks Gambling Screen (SOGS; Lesieur and Blume, 1987)

This questionnaire uses 20 items to assess cognitive, emotional, and behavioral aspects related to problem gambling by measuring the severity of gambling activity (responses ranging from 0 to 20). This questionnaire discriminates between non-problem gambling (from 0 to 2), light problem gambling (from 3 to 4), and problem gambling (from 5 to 20), with higher scores being indicative of greater gambling severity. The SOGS is the most commonly used instrument to evaluate gambling severity in research and clinical setting, though it was designed to only be administered to adults (Jiménez-Murcia et al., 2016; Mestre-Bach et al., 2016). The validation of the Spanish SOGS showed high internal consistency (*Cronbach’s alpha* = 0.94) and good test–retest reliability (*r* = 0.98) (Echeburúa et al., 1994). In the present study, this questionnaire was administered as a measure of convergent validity for the CAGI.

### Procedure

The CAGI was translated into Spanish in accordance with the International Test Commission Guidelines for Translating and Adapting Tests (International Test Commission [ITC], 2010). Two bilingual clinical psychologists with extensive experience in GD translated the original English version into Spanish. This translated Spanish version of the CAGI was then back-translated and any differences between the original and back-translated versions were discussed and resolved by consensus. The Spanish CAGI was reviewed by two other Spanish-speaking clinical psychologists, who had not been involved in the back-translation procedure. This was done in order to confirm that the instrument was clear and understandable for younger populations. The Spanish version of the CAGI is available in Supplementary Data Sheet 1.

The assessment was conducted prospectively in a single session (mean duration of 90 min), during which the abovementioned tests were administered by trained clinical psychologists from the Unit staff. In addition to the assessment battery, patients underwent a semi-structured face-to-face interview regarding their gambling behavior and psychopathological symptoms (Jiménez-Murcia et al., 2006). This interview also gathered sociodemographic data (e.g., education, occupation, marital status) and additional clinical information.

### Statistical Methods

Stata 13.1 for Windows was used to conduct statistical analyses. Firstly, the dimensional-structure of the CAGI was analyzed through confirmatory factor analysis (CFA) and exploratory factor analysis (EFA). Due to the asymmetrical distribution of the questionnaire scores in the control group (many items were registered in the negative range), the factorials procedures were run selecting only the GD group (whose responses registered more symmetrical distributions). For the CFA, overall goodness-of-fit statistics were assessed through the χ^2^ test, the root mean square error of approximation (RMSEA), baseline comparison indexes (comparative fit index, CFI and Tucker-Lewis index, TLI), and the size of residuals (standardized root mean square residual, SRMR). A fit was considered to be good if a non-significant result (*p* > 0.05) was achieved for the χ^2^ test, the RMSEA was lower than 0.08, the CFI/TLI coefficients were higher than 0.90, and the SRMR was limited to 0.08 (Kline, 2010). For the EFA, the adequacy of sampling was valued with the Kaiser-Meyer-Olkin test (KMO) (which reports a measure of how suited the empirical data is for factor analysis; values between 0.80 and 1.00 are considered good, 0.70–0.79 acceptable, 0.60–0.70 fair and lower than 0.60 inadequate) and the Bartlett’s test of sphericity (a measure of the validity and suitability of the data to the factor; *p* < 0.05 is considered adequate). The internal consistency of derived scale(s) obtained in the factor analysis was measured by Cronbach’s alpha (α-coefficient), considering α > 0.80 to be adequate. The best factor solution was determined by several considerations (Tabachnick and Fidell, 2001): (a) factors with adequate clinical interpretability; (b) Kaiser’s criterion (factors with eigenvalues over 1, results of the scree plot and a total variance explained of at least 30%); (c) factors with an adequate number of items (the higher the number of items, the greater the reliability); and (d) no high correlations between the factors.

Secondly, the best cut-off for the CAGI score to discriminate between GD cases and controls was selected with ROC methodology, which is usually employed in clinical epidemiology to quantify how accurately medical diagnostic tests (or systems) can discriminate between patient states, typically referred to as *diseased* and *non-diseased*. In this work, the ROC method was used to obtain the Area Under the ROC curve (AUC), which represents a global measure of the validity of the diagnostic capacity of the CAGI across all the cut-off points. Next, since the best cut-off point depends on the disorder prevalence and the cost/risk for correct and false diagnoses (Zhou et al., 2002), the ROC analysis was performed considering different scenarios with different disease prevalence and diverse cost/risk for correct and false classifications.

Finally, the convergent validity of the CAGI with an external measure of the problem gambling severity (SOGS) was estimated by means of Pearson’s *r*, with |*r*| ≥ 0.30 considered evidence of relevant association (Cicchetti, 1994).

## Results

**Table 1** displays the demographic characteristics of the two groups.

**Table 1:** Demographic characteristics of samples.

	Total (*n* = 395)	Control (*n* = 340)	Clinical (*n* = 55)	Statistic	df	*p*
Age (years);						
*mean (SD)*	21.9 (3.59)	21.4 (3.30)	25.2 (3.55)	*t* = 7.86	393	<0.001
Gender; %						
*Males*	73.9	69.7	100	χ^2^ = 22.54	1	<0.001
Education; %						
*Primary or less*	22.3	13.8	72.7	χ^2^ = 93.92	2	<0.001
*Secondary*	63.2	70.2	21.8			
*University*	14.5	16.0	5.45			
Marital status; %						
*Single*	91.3	93.6	77.8	χ^2^ = 14.51	1	<0.001
*Married/with partner*	8.7	6.39	22.2			
Employment status; %						
*Employed*	31.0	28.0	47.3	χ^2^ = 8.07	1	0.004

*SD, standard deviation.*

The two subsamples differed on all demographic variables (See **Table 1**). The clinical group was older, had more males, was less educated, was more likely to be married and employed, than the control group.

The initial CFA carried out in the GD group did not obtain goodness-of-fit to verify the original structure of the CAGI: χ^2^ = 285.4 (*p* < 0.001), RMSEA = 0.108 (95% confidence interval: 0.083–0.132), CFI = 0.730, TLI = 0.690, SRMR = 0.110. The strong associations between factors (ranging between *r* = 0.63 and *r* = 0.87) suggested that a solution with a low number of factors should constitute a preferable structure.

Since no additional empirical research was available to test alternative factor structures for Spanish data, EFA was used and factor-components were extracted with both oblique rotation (oblimin procedure) and non-oblique rotation (varimax method). Supplementary Table S1 shows results for the different candidate solutions obtained in the EFA run in the GD subsample (*n* = 55). The sampling adequacy test for this analysis obtained adequate results: KMO = 0.710 and Bartlett’s Test of Sphericity *p* < 0.001. Based on the set of results, the one-dimension solution was considered an optimal solution: the percentage of explained variance was adequate (31.4%), the scree plot indicated that from the one dimension, each successive factor accounted for smaller amounts of the total variance (see **Supplementary Figure S1**), solutions with more than one dimension tended to obtain high inter-correlations between the factors or factors with a low number of items, and solutions with more than one dimension tended to be clinically more difficult to interpret. Therefore, the one-factor model was selected as the best solution, with an excellent internal consistency (α = 0.91). **Table 2** shows factor loadings of this final one-factor solution. Supplementary Table S2 includes the distribution of the CAGI 24-item raw score for the control and clinical groups, stratified by age. No differences in the mean scores in the control group were due to sex (*p* = 0.159) or age (*p* = 0.091), and nor in the GD group did differences related to age either emerge (*p* = 0.567).

**Table 2:** Factor loadings for the exploratory factor analysis (EFA) in the one-factor solution.

21. Feel guilty	0.558
22. Skip practice or drop out of activities	0.597
23. Feel sad/depressed	0.647
24. Skip family gatherings	0.561
25. Feel frustrated	0.578
26. Skip hanging out with friends	0.640
27. Plan gambling/betting activities	0.613
28. Feel bad	0.557
29. Skip get-togethers with friends	0.684
30. Gamble/bet the winnings	0.504
31. Feel stressed	0.642
32. Others complain he/she gambles too much	0.555
33. Gamble/bet for long periods of time	0.705
34. Feel it would be better to stop gambling	0.256
35. Go back another day to try to win	0.660
36. Gamble/bet with a lot of money	0.691
37. Hide gambling/bets from others	0.466
38. Have problems paying gambling bets	0.571
39. Receive pressure to pay bets	0.486
40. Feel that gambling/betting is a problem	0.430
41. Borrow money from others	0.446
42. Take money from lunch/clothing allowance, etc.	0.561
43. Sell personal property	0.408
44. Steal money in order to gamble/bet	0.321

The CAGI 24-item total score obtained very good validity as a screening tool for GD. The AUC, which measures the discrimination ability (accuracy) of the test to correctly classify those subjects with and without the disease, was excellent (AUC = 0.99, *SE* = 0.011, *p* < 0.001) (the ROC curve is plotted in **Supplementary Figure S2**; an area of 0.50 is marked by the discontinuous line representing a worthless test, while an area of 1 represents a perfect test). The most accurate cut-score for this measure was 11 or more (hit rate = 0.99, sensitivity Se = 0.98 and specificity Sp = 0.99). Supplementary Table S3 contains the complete ROC results obtained for hypothetical scenarios defined for different costs/risks for a false negative screen compared to a false positive screen and GD prevalence. For example, considering a GD prevalence equal to 20% and one-third cost for a false negative screen compared with a false positive screen (ratio = 1/3 in Supplementary Table S3), the best cut-off for the CAGI 24-item is 16 (Se = 92.5%; Sp = 100.0).

In this study, the classification accuracy of the CAGI 9-item GPSS, using the standard cut-score score of 6 or more, was very good, with hit rate = 0.98, Se = 0.93, and Sp = 0.99, and similar to the accuracy achieved by the CAGI 24-item scale (see Supplementary Table S4).

Convergent validity between the CAGI 24-item and the SOGS total score was very good: *r* = 0.33 for control group and *r* = 0.74 for clinical group.

## Discussion

This study aimed to translate and validate the CAGI questionnaire into the Spanish language for its use in the Spanish young population. We also sought to assess the psychometric soundness of this questionnaire. The paucity of instruments examining gambling behavior in young populations is a noteworthy shortcoming in the literature and our aim was to address this gap by validating the CAGI, an instrument specifically designed with adolescents in mind. The CAGI is unique in that the questionnaire items were designed in a developmentally sensitive manner and cover issues that are particular to adolescents (e.g., social pressures, parents’ money, etc.).

Given that significant sociodemographic differences were found between the control and GD subgroups in our samples, we opted to conduct our CFA only in the GD group. Factor analysis yielded the best fit for the one-factor model and excellent internal consistency. Evidence of convergent validity between the CAGI and alternate measure of problem gambling severity (high correlation between CAGI and SOGS scores for the entire sample, however, lower correlations within each subsample) was in the good to very good range. It should be noted that the SOGS was designed for adult gamblers, though its use in all types of populations is widespread (Lesieur and Blume, 1987; Echeburúa et al., 1994). Finally, the classification accuracy of the GPSS subscale was excellent.

### Limitations

Limitations of this study are primarily due to sample characteristics. There are very few young adolescents who seek GD treatment and who are available to recruit for this type of study. This was the case for the CAGI development study (Tremblay et al., 2010a) as well as for this study. Therefore, in order to recruit a clinical group of sufficient size, it was necessary to include young adults, in their late teens and 20s. This also resulted in a relatively small sample size for the clinical group (*n* = 55) in this study and this could limit the range of variability of the item responses and consequently, the emergence of an internal structure with a higher number of dimensions. Relatedly, the inclusion of young adult participants is a limitation in that it exceeds the age range used in the development of the CAGI and may introduce age as a confounder when comparing results, and it could also be an additional reason for obtaining a different factorial structure. The two groups differed in all demographic variables and this introduced confounds. Future research will need to attempt to recruit larger clinical and control subsamples that have similar demographic characteristics in order to eliminate these demographic differences.

## Conclusion

The CAGI is one of the few instruments specifically developed for the assessment of problem gambling among adolescents and young people. This study upholds its psychometric robustness in assessing gambling behavior in the Spanish population. It is a reliable, valid, accurate instrument that can provide a global severity score for problem gambling in young people. The systematic application of this measure in high-risk populations would enable early identification of those cases most vulnerable to the development of a GD, thereby allowing intervention and prevention programs to be targeted where they are most needed.

## Ethics Statement

The Ethics Committee of Bellvitge University Hospital (Barcelona) This study was carried out in accordance with the latest version of the Declaration of Helsinki. The Ethics Committee of Bellvitge University Hospital (Barcelona) approved the study, and signed informed consent was obtained from all final participants.

## Author Contributions

SJ-M, RG, FF-A, JM, RS, and JT contributed to the design of the work, acquisition, and interpretation of the data. RG, ST, VM-R, GM-B, and TS were responsible for statistical analysis and for writing statistical sections of the manuscript. AdP-G, LM, LS, KG, NA, and MG-P contributed to administering, correcting, and entering questionnaires in the database. RG, SJ-M, ST, and VM-R contributed to interpreting the psychological tests of this study. GM-B, RS, TS and, SJ-M were responsible for the revision of the article and addressing the reviewer’s needs. All authors contributed to critically revising the work, approved the final version of the article to be published and agreed to be accountable for all aspects of the work in ensuring that questions related to the accuracy or integrity of any part of the work are appropriately investigated and resolved.

## Conflict of Interest Statement

The authors declare that the research was conducted in the absence of any commercial or financial relationships that could be construed as a potential conflict of interest.

## Supplementary material

FIGURE S1 **Scree plot obtained in the EFA analysis (GD group, *n* = 55)**.

FIGURE S2 **CAGI discrimination ability (accuracy).** The ROC curve is plotted with an area of 0.50 is marked by the discontinuous line representing a worthless test, while an area of 1 represents a perfect test.
