# Supplementary figures and images for: A Spanish Validation of the Canadian Adolescent Gambling Inventory (CAGI)
Source: Front Psychol. 2017 Feb 7;8:177. doi: 10.3389/fpsyg.2017.00177 (PMC5293835; doi:10.3389/fpsyg.2017.00177)

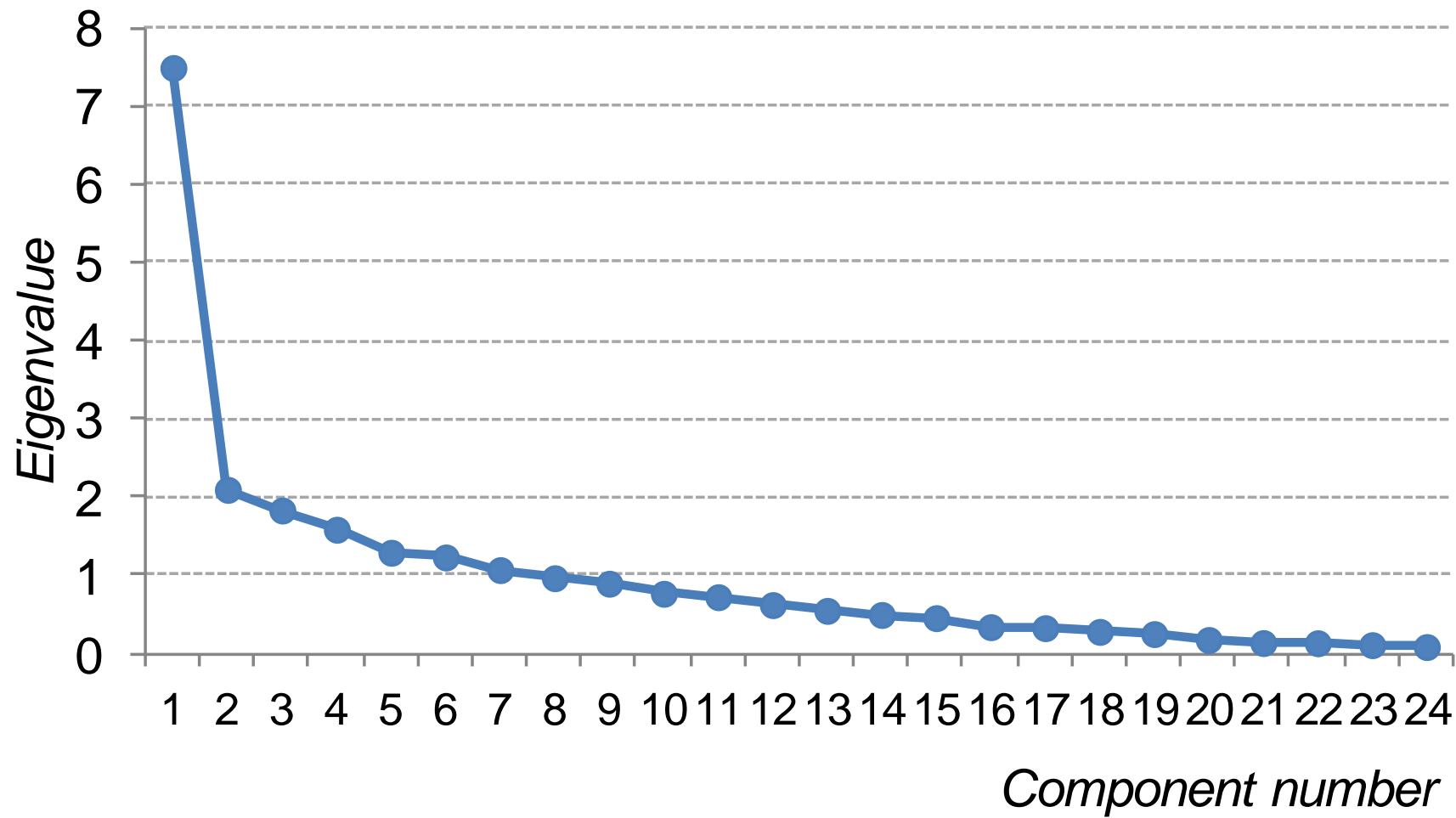

Figure S1 (supplementary). Scree plot obtained in the EFA analysis (GD group, n=55).

Supplement: FIGURE S1 — Scree plot obtained in the EFA analysis (GD group, n = 55). [file Image_1.PDF]

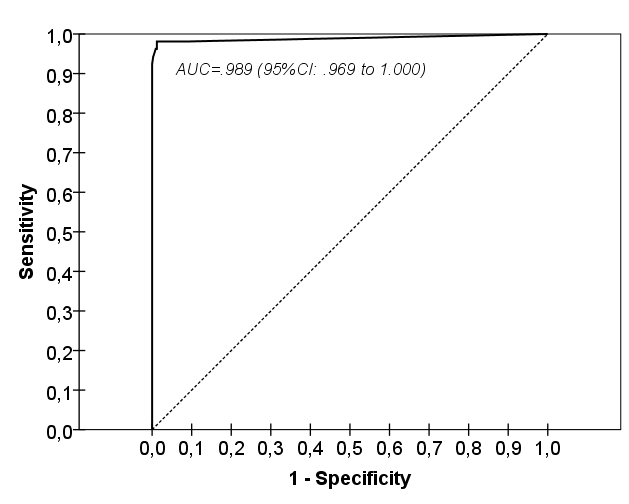

Supplement: FIGURE S2 — CAGI discrimination ability (accuracy). The ROC curve is plotted with an area of 0.50 is marked by the discontinuous line representing a worthless test, while an area of 1 represents a perfect test. [file Image_2.PNG]
