# Supplementary material for: A Spanish Validation of the Canadian Adolescent Gambling Inventory (CAGI)
Source: Front Psychol. 2017 Feb 7;8:177. doi: 10.3389/fpsyg.2017.00177 (PMC5293835; doi:10.3389/fpsyg.2017.00177)
Supplement: Supplementary file 3 [file Table_1.DOCX]

Table S1. Factor loadings for the exploratory factor analysis.

|  |  | Orthogonal rotation: Varimax | | | | | | | | | Oblique rotation: Oblimin | | | | | | | | |
| --- | --- | --- | --- | --- | --- | --- | --- | --- | --- | --- | --- | --- | --- | --- | --- | --- | --- | --- | --- |
| Solution: number of factors | 1-Factor | 2-Factors | | 3-Factors | | | 4-Factors | | | | 2-Factors | | 3-Factors | | | 4-Factors | | | |
| Item | F1 | F1 | F2 | F1 | F2 | F3 | F1 | F2 | F3 | F4 | F1 | F2 | F1 | F2 | F3 | F1 | F2 | F3 | F4 |
| 21. Feel guilty | **.558** | .243 | **.547** | **.541** | .070 | .380 | .286 | .058 | **.700** | .019 | .138 | **.527** | -.053 | **.547** | .297 | -.079 | .185 | **.705** | -.055 |
| 22. Skip practice or drop out of activities | **.597** | **.544** | .299 | .255 | **.560** | .184 | .316 | **.518** | .102 | .233 | **.518** | .187 | **.533** | .172 | .086 | **.445** | .267 | .026 | .205 |
| 23. Feel sad/depressed | **.647** | .330 | **.586** | **.572** | .178 | .381 | .345 | .158 | **.679** | .051 | .222 | **.548** | .055 | **.562** | .282 | .013 | .241 | **.668** | -.025 |
| 24. Skip family gatherings | **.561** | **.613** | .178 | .116 | **.811** | -.059 | .198 | **.798** | .049 | -.095 | **.621** | .040 | **.839** | -.002 | -.170 | **.787** | .154 | .028 | -.138 |
| 25. Feel frustrated | **.578** | **.455** | .362 | .337 | .312 | **.384** | .131 | .322 | **.618** | .032 | **.408** | .276 | .245 | .287 | **.305** | .223 | .014 | **.636** | -.038 |
| 26. Skip hanging out with friends | **.640** | **.778** | .125 | .066 | **.727** | .333 | .056 | **.732** | .265 | .209 | **.811** | -.059 | **.742** | -.068 | .248 | **.684** | -.050 | .242 | .166 |
| 27. Plan gambling/betting activities | **.613** | **.634** | .231 | .187 | **.540** | .359 | .205 | **.513** | .195 | .355 | **.631** | .091 | **.517** | .091 | .277 | **.433** | .125 | .127 | .327 |
| 28. Feel bad | **.557** | **.615** | .171 | .138 | .396 | **.520** | -.093 | .439 | **.665** | .101 | **.625** | .031 | .367 | .050 | **.465** | .363 | -.246 | **.711** | .033 |
| 29. Skip get-togethers with friends | **.684** | **.793** | .172 | .110 | **.782** | .289 | .166 | **.764** | .148 | .277 | **.817** | -.012 | **.793** | -.027 | .189 | **.709** | .079 | .090 | .243 |
| 30. Gamble/bet the winnings | **.504** | .040 | **.676** | **.665** | .168 | -.089 | **.691** | .075 | .119 | -.009 | -.111 | **.716** | .047 | **.695** | -.212 | -.042 | **.714** | .023 | -.046 |
| 31. Feel stressed | **.642** | **.623** | .284 | .236 | **.601** | .262 | .171 | **.597** | .368 | .073 | **.608** | .150 | **.577** | .140 | .163 | **.527** | .075 | .354 | .016 |
| 32. Others complain he/she gambles too much | **.555** | .348 | **.436** | **.409** | .363 | .140 | **.353** | .328 | .310 | .026 | .277 | **.382** | .295 | **.372** | .039 | .238 | **.301** | .272 | -.024 |
| 33. Gamble/bet for long periods of time | **.705** | .325 | **.674** | **.650** | .330 | .173 | **.594** | .260 | .360 | .104 | .197 | **.644** | .210 | **.635** | .040 | .118 | **.549** | .280 | .048 |
| 34. Feel it would be better to stop gambling | .256 | -.175 | **.541** | **.564** | -.282 | .153 | **.435** | -.336 | .334 | .078 | -.311 | **.624** | -.420 | **.642** | .102 | -.461 | **.427** | .285 | .049 |
| 35. Go back another day to try to win | **.660** | .164 | **.772** | **.753** | .274 | -.012 | **.796** | .165 | .146 | .098 | .002 | **.789** | .138 | **.768** | -.159 | .020 | **.806** | .020 | .057 |
| 36. Gamble/bet with a lot of money | **.691** | .188 | **.792** | **.778** | .208 | .121 | **.752** | .109 | .292 | .145 | .022 | **.805** | .057 | **.797** | -.019 | -.054 | **.736** | .179 | .096 |
| 37. Hide gambling/bets from others | **.466** | .172 | **.488** | **.482** | .081 | .235 | .335 | .052 | **.455** | .040 | .074 | **.482** | -.022 | **.491** | .157 | -.062 | .275 | **.432** | -.012 |
| 38. Have problems paying gambling bets | **.571** | .306 | **.503** | **.472** | .419 | -.003 | **.535** | .347 | .079 | .078 | .215 | **.465** | .348 | **.441** | -.123 | .256 | **.528** | -.010 | .044 |
| 39. Receive pressure to pay bets | **.486** | .248 | **.440** | .401 | **.529** | -.260 | **.530** | .460 | -.081 | -.139 | .168 | **.411** | **.492** | .365 | -.388 | .415 | **.557** | -.154 | -.172 |
| 40. Feel that gambling/betting is a problem | **.430** | **.367** | .240 | .237 | .012 | **.636** | .008 | .026 | **.609** | .349 | **.342** | .167 | -.065 | .210 | **.612** | -.091 | -.118 | **.606** | .308 |
| 41. Borrow money from others | **.446** | **.433** | .197 | .176 | .258 | **.403** | .276 | .199 | -.033 | **.634** | **.422** | .104 | .219 | .121 | **.355** | .104 | .241 | -.163 | **.648** |
| 42. Take money from lunch/clothing allowance | **.561** | .276 | **.519** | **.500** | .255 | .176 | **.550** | .172 | .100 | .309 | .180 | **.489** | .161 | **.486** | .074 | .050 | **.536** | -.018 | .289 |
| 43. Sell personal property | **.408** | **.530** | .045 | .030 | .141 | **.713** | .018 | .122 | .180 | **.798** | **.562** | -.083 | .110 | -.037 | **.708** | .020 | -.076 | .085 | **.810** |
| 44. Steal money in order to gamble/bet | **.321** | **.434** | .018 | .010 | .070 | **.645** | .025 | .046 | .093 | **.782** | **.464** | -.088 | .043 | -.044 | **.649** | -.046 | -.048 | -.006 | **.804** |
| Explained variance for each factor (%) | 31.24 | 31.24 | 8.75 | 31.24 | 8.75 | 7.65 | 31.24 | 8.75 | 7.65 | 6.62 | 31.24 | 8.75 | 31.24 | 8.75 | 7.65 | 31.24 | 8.75 | 7.65 | 6.62 |
| Explained variance for the model (%) | 31.24 | 39.99 |  | 47.63 |  |  | 54.26 |  |  |  | 39.99 |  | 47.63 |  |  | 54.26 |  |  |  |
| Cronbach’s alpha (α) | .908 | .846 | .855 | .850 | .839 | .708 | .832 | .836 | .771 | .707 | .846 | .855 | .839 | .850 | .708 | .836 | .832 | .771 | .707 |
| Correlations between factors; F1-F2 |  | .577 |  | .566 |  |  | .516 |  |  |  | .577 |  | .566 |  |  | .516 |  |  |  |
| F1-F3 |  |  |  | .485 |  |  | .536 |  |  |  |  |  | .507 |  |  | .518 |  |  |  |
| F1-F4 |  |  |  |  |  |  | .302 |  |  |  |  |  |  |  |  | .354 |  |  |  |
| F2-F3 |  |  |  | .507 |  |  | .518 |  |  |  |  |  | .485 |  |  | .536 |  |  |  |
| F2-F4 |  |  |  |  |  |  | .354 |  |  |  |  |  |  |  |  | .302 |  |  |  |
| F3-F4 |  |  |  |  |  |  | .305 |  |  |  |  |  |  |  |  | .305 |  |  |  |

*Note.* Bold: high factor loading (>.30) and selected in the factor. Analysis for the ED group, *n*=55.
