# Supplementary material for: A Spanish Validation of the Canadian Adolescent Gambling Inventory (CAGI)
Source: Front Psychol. 2017 Feb 7;8:177. doi: 10.3389/fpsyg.2017.00177 (PMC5293835; doi:10.3389/fpsyg.2017.00177)
Supplement: Supplementary file 4 [file Table_2.DOCX]

Table S2. Distribution of the CAGI total scores.

| Percentiles → | 5 | 10 | 25 | 50 | 75 | 80 | 85 | 90 | 91 | 92 | 93 | 94 | 95 | 96 | 97 | 98 | 99 | Mean | SD |
| --- | --- | --- | --- | --- | --- | --- | --- | --- | --- | --- | --- | --- | --- | --- | --- | --- | --- | --- | --- |
| Control; *n*=340 | - | - | - | 0 | 0 | 0 | 0 | 0 | 1 | 1 | 1 | 2 | 3 | 5 | 7 | 8 | 13 | 0.45 | 1.93 |
| Age: 16-20; *n*=161 | - | - | - | 0 | 0 | 0 | 0 | 0 | 0 | 1 | 1 | 2 | 3 | 4 | 5 | 8 | 11 | 0.37 | 1.62 |
| Age: 21-25; *n*=132 | - | - | - | 0 | 0 | 0 | 0 | 0 | 1 | 2 | 3 | 6 | 7 | 8 | 9 | 13 | 14 | 0.70 | 2.52 |
| Age: 26-30; *n*=47 | - | - | - | 0 | 0 | 0 | 0 | 0 | 0 | 0 | 0 | 0 | 0 | 1 | 2 | 2 | - | 0.02 | 0.15 |
| GD; *n*=55 | 12 | 18 | 33 | 41 | 49 | 52 | 56 | 59 | 60 | 60 | 60 | 61 | 61 | 62 | 62 | 62 | - | 39.8 | 13.7 |
| Age: 16-20; *n*=9 | 0 | 0 | 33 | 39 | 42 | 43 | 52 | - | - | - | - | - | - | - | - | - | - | 36.3 | 16.0 |
| Age: 21-25; *n*=13 | 11 | 16 | 33 | 45 | 56 | 59 | 60 | 61 | 62 | 63 | 63 | - | - | - | - | - | - | 42.8 | 14.7 |
| Age: 26-30; *n*=33 | 14 | 18 | 33 | 40 | 50 | 53 | 55 | 56 | 57 | 58 | 59 | 60 | 61 | 62 | - | - | - | 39.7 | 12.8 |

*Note.* GD: gambling disorder.
