# Supplementary material for: A Spanish Validation of the Canadian Adolescent Gambling Inventory (CAGI)
Source: Front Psychol. 2017 Feb 7;8:177. doi: 10.3389/fpsyg.2017.00177 (PMC5293835; doi:10.3389/fpsyg.2017.00177)
Supplement: Supplementary file 5 [file Table_3.DOCX]

Table S3. Selection of the best cut-off for the CAGI considering different hypothetical scenarios.

|  |  | Ratio of a false negative screening cost compared with false positive screening cost | | | | | | | | |
| --- | --- | --- | --- | --- | --- | --- | --- | --- | --- | --- |
| GD prevalence |  | 1/5 | 1/4 | 1/3 | 1/2 | 1 | 2 | 3 | 4 | 5 |
| 5% | Best cut-off | 16 | 16 | 16 | 16 | 16 | 16 | 15 | 15 | 11 |
|  | Sensitivity (%) | 92.5 | 92.5 | 92.5 | 92.5 | 92.5 | 92.5 | 94.3 | 94.3 | 98.1 |
|  | Specificity (%) | 100 | 100 | 100 | 100 | 100 | 100 | 99.7 | 99.7 | 98.8 |
| 10% | Best cut-off | 16 | 16 | 16 | 16 | 16 | 15 | 11 | 11 | 11 |
|  | Sensitivity (%) | 92.5 | 92.5 | 92.5 | 92.5 | 92.5 | 94.3 | 98.1 | 98.1 | 98.1 |
|  | Specificity (%) | 100 | 100 | 100 | 100 | 100 | 99.7 | 98.8 | 98.8 | 98.8 |
| 20% | Best cut-off | 16 | 16 | 16 | 16 | 11 | 11 | 11 | 11 | 11 |
|  | Sensitivity (%) | 92.5 | 92.5 | 92.5 | 92.5 | 98.1 | 98.1 | 98.1 | 98.1 | 98.1 |
|  | Specificity (%) | 100 | 100 | 100 | 100 | 98.8 | 98.8 | 98.8 | 98.8 | 98.8 |
| 30% | Best cut-off | 16 | 16 | 16 | 15 | 11 | 11 | 11 | 11 | 11 |
|  | Sensitivity (%) | 92.5 | 92.5 | 92.5 | 94.3 | 98.1 | 98.1 | 98.1 | 98.1 | 98.1 |
|  | Specificity (%) | 100 | 100 | 100 | 99.7 | 98.8 | 98.8 | 98.8 | 98.8 | 98.8 |
| 40% | Best cut-off | 16 | 15 | 15 | 11 | 11 | 11 | 11 | 11 | 11 |
|  | Sensitivity (%) | 92.5 | 94.3 | 94.3 | 98.1 | 98.1 | 98.1 | 98.1 | 98.1 | 98.1 |
|  | Sensitivity (%) | 100 | 99.7 | 99.7 | 98.8 | 98.8 | 98.8 | 98.8 | 98.8 | 98.8 |
| 50% | Specificity (%) | 15 | 11 | 11 | 11 | 11 | 11 | 11 | 11 | 11 |
|  | Sensitivity | 94.3 | 98.1 | 98.1 | 98.1 | 98.1 | 98.1 | 98.1 | 98.1 | 98.1 |
|  | Specificity | 99.7 | 98.8 | 98.8 | 98.8 | 98.8 | 98.8 | 98.8 | 98.8 | 98.8 |

*Note.* GD: gambling disorder. *N*=395.
