# Supplementary material for: A Spanish Validation of the Canadian Adolescent Gambling Inventory (CAGI)
Source: Front Psychol. 2017 Feb 7;8:177. doi: 10.3389/fpsyg.2017.00177 (PMC5293835; doi:10.3389/fpsyg.2017.00177)
Supplement: Supplementary file 6 [file Table_4.DOCX]

Table S4 (supplementary). Screening accuracy for the CAGI 9-item GPSS subscale and the CAGI 24-item scale in the sample (*N=*395, base rate=13.9%).

|  | Cut-off score | HR | (95% CI HR) | Se | 95%CI | Sp | 95%CI |
| --- | --- | --- | --- | --- | --- | --- | --- |
| CAGI 9-item GPSS | 6 | 98.46 | (97.24 to 99.68) | 92.59 | (85.61 to 99.58) | 99.40 | (98.58 to 1.00) |
| CAGI 24-item full scale | 11 | 98.71 | (97.60 to 99.83) | 98.11 | (94.45 to 1.00) | 98.81 | (97.65 to 99.97) |

*Note.* HR: hit rate (percent, %). Se: sensitivity (percent, %). Sp: specificity (percent, %).
