# Supplementary material for: A Spanish Validation of the Canadian Adolescent Gambling Inventory (CAGI)
Source: Front Psychol. 2017 Feb 7;8:177. doi: 10.3389/fpsyg.2017.00177 (PMC5293835; doi:10.3389/fpsyg.2017.00177)
Supplement: Supplementary file 7 [file Data_Sheet_1.PDF]

CAGI

Inventario Canadiense de Juego para Adolescentes, V 1.09, mayo 2010

(Tremblay, J., Wiebe, J., Stinchfield, R., Wynne, H., 2010. Adaptación española a cargo de de Jiménez-Murcia, S., Stinchfield, R., & Tremblay, J., 2011)

Fecha (Año/Mes/Día): **Edad:** **Sexo: Hombre/Mujer**

Nivel de estudios: ESO/Bachillerato/FPI/FPII/Estudios universitarios/Otros

Nombre (opcional):
Apellidos (opcional):

El siguiente cuestionario trata sobre el juego. Por juego, entendemos el hecho de apostar o arriesgar dinero u objetos de valor con la finalidad de ganar dinero o cualquier otra cosa de valor.

1. EN LOS ÚLTIMOS TRES MESES…
¿Con qué frecuencia ha apostado o jugado dinero u objetos de valor en las siguientes actividades y, aproximadamente, cuánto tiempo por semana ha destinado en cada una?

1a) ¿Con qué frecuencia ha jugado o apostado en esta actividad? Si usted ha contestado "No en los últimos 3 meses", vaya a la siguiente actividad.

RESPUESTAS: No en los últimos 3 meses/1 vez al mes/2-3 veces al mes/1 vez a la semana/2-6 veces a la semana/Diariamente

1b) En los últimos tres meses, aproximadamente, ¿cuánto tiempo ha destinado en esta actividad en una semana normal (horas: minutos)?

1. Internet (con dinero)

Póker Máquinas tragaperras Otros

Los siguientes ítems no incluyen Internet

2. Cupones de lotería (p.ej., 6/49, Súper 7)
3. Cupones Rascas o de premio instantáneo
4. Rifas o cupones para recaudar fondos
5. Blotzito
6. Juegos de cartas con dinero (Póker, Black Jack, etc.)
7. Tableros o dados (con dinero)
8. Centros de lotería visionados
9. Máquinas tragaperras en casinos o circuitos
10. Videojuegos con dinero o objetos de valor
11. Apuestas deportivas por Internet - Sport Select (p.ej., Pro Line, Over/Under, Point Spread)
12. Apuestas o juegos deportivos (esto es, hockey, baloncesto, etc.)
13. Deportes mediante un corredor de apuestas (esto es, alguien que acepta y paga las apuestas)
14. Carreras de caballos
15. Juegos de mesa en casinos (p.ej., póker, Black Jack, ruleta,etc.)
16. Rendimiento propio o de alguien en juegos de destreza (p.ej., billar, golf, bolos, dardos) u otras actividades
17. Un desafío o reto sobre que usted o alguien puede hacer algo
18. Bingo (con dinero o con objetos de valor)
19. Otras formas de juego/apuesta (¿qué es? Por favor, escríbalo)

Si usted no ha jugado en ninguna de las actividades anteriores DURANTE LOS TRES ÚLTIMOS MESES, el cuestionario está finalizado. Si usted ha jugado en una o más actividades, por favor continúe en el ítem 20a.

20a. DURANTE LOS TRES ÚLTIMOS MESES, ¿cuánto dinero, en total, ha perdido jugando/apostando? Si no ha perdido dinero, escriba “0” (en euros)
20b. DURANTE LOS TRES ÚLTIMOS MESES, ¿ha perdido algún objeto de valor por jugar/apostar? Si la respuesta es Sí, escriba su valor (en euros), y cuál fue ese objeto (si han sido más de un objeto, calcule el valor total):

Las siguientes preguntas hacen referencia a su conducta de juego/apuesta SOBRE LOS ÚLTIMOS TRES MESES.

21. ¿Con qué frecuencia se ha sentido culpable de la cantidad de dinero que ha perdido en su conducta de juego/apuesta?
22. ¿Con qué frecuencia ha faltado a clase o ha abandonado actividades (tales como deportes en equipo o grupo de amistades) debido a su conducta de juego/apuesta?

23. ¿Con qué frecuencia se ha sentido triste o deprimido por el hecho de haber perdido dinero en el juego/apuesta?
24. ¿Con qué frecuencia ha faltado a eventos familiares para poder jugar/apostar?
25. ¿Con qué frecuencia el juego/apuesta le ha hecho sentir frustrado?
26. ¿Con qué frecuencia ha evitado pasar tiempo con amistades que no juegan/apuestan para poder pasar tiempo con amistades que sí juegan/apuestan?
27. ¿Con qué frecuencia ha planeado sus actividades de juego/apuesta?
28. ¿Con qué frecuencia se ha sentido mal por la manera cómo juega/apuesta o qué sucede cuando juega/apuesta?
29. ¿Con qué frecuencia ha evitado asistir a reuniones con amigos para poder jugar/apostar?
30. ¿Con qué frecuencia juega/apuesta sus ganancias?
31. ¿Con qué frecuencia el juego/apuesta he la hecho sentir estresado?
32. ¿Con qué frecuencia sus familiares o amigos se han quejado sobre el hecho que usted juega demasiado?

RESPUESTAS: Nunca/A veces/A menudo/Casi siempre

Las siguientes preguntas hacen referencia a su conducta de juego/apuesta SOBRE LOS ÚLTIMOS TRES MESES.

33. ¿Con qué frecuencia ha jugado/apostado durante períodos de tiempo más largos de lo que tenía planeado?
34. ¿Con qué frecuencia ha sentido que sería mejor para su bienestar personal parar de apostar/jugar?
35. ¿Con qué frecuencia ha vuelto otro día para intentar recuperar el dinero que había perdido en el juego/apuesta?
36. ¿Con qué frecuencia ha jugado/apostado más dinero del que usted tenía planeado?
37. ¿Con qué frecuencia ha ocultado su conducta de juego/apuesta a sus padres, otros miembros de la familia o profesores?
38. ¿Con qué frecuencia ha tenido dificultades para pagar sus deudas relacionadas con su conducta de juego?
39. ¿Con qué frecuencia alguien ha tenido que presionarle, independientemente del modo, para pagarle lo que usted le debía después de haber perdido un juego/apuesta?
40. En los últimos tres meses, ¿con qué frecuencia ha sentido que es posible que usted puede tener un problema con el juego/apuesta?

RESPUESTAS: Nunca/A veces/A menudo/Casi siempre

A veces las personas hacen cosas para apostar/jugar. Por favor, indíquenos con qué frecuencia ha hecho las siguientes cosas en LOS ÚLTIMOS 3 MESES:

41. ¿Con qué frecuencia ha cogido dinero prestado de su familia, amigos u otros para apostar/jugar?
42. ¿Con qué frecuencia ha cogido dinero que iba a gastarse en comida, ropa, películas, etc., pero luego lo ha usado para jugar/apostar o para pagar deudas debidas al juego?

43. ¿Con qué frecuencia ha vendido sus propiedades personales (tales como objetos electrónicos, ropa, etc.) para tener dinero para jugar/apostar o bien para pagar sus deudas de juego?
44. ¿Con qué frecuencia ha robado dinero u otros objetos de valor para poder jugar/apostar o para pagar sus deudas relacionadas con el juego/apuesta?

RESPUESTAS: Nunca/1-3 veces/4-6 veces/7 o más veces
